# IL4Rα Signaling Abrogates Hypoxic Neutrophil Survival and Limits Acute Lung Injury Responses *In Vivo*

**DOI:** 10.1164/rccm.201808-1599OC

**Published:** 2019-07-15

**Authors:** Alison J. Harris, Ananda S. Mirchandani, Ruairi W. Lynch, Fiona Murphy, Liam Delaney, Donna Small, Patricia Coelho, Emily R. Watts, Pranvera Sadiku, David Griffith, Rebecca S. Dickinson, Eilidh Clark, Joseph A. Willson, Tyler Morrison, Massimilliano Mazzone, Peter Carmeliet, Bart Ghesquiere, Cecilia O’Kane, Danny McAuley, Steve J. Jenkins, Moira K. B. Whyte, Sarah R. Walmsley

**Affiliations:** ^1^ Medical Research Council/University of Edinburgh Centre for Inflammation Research, Queen’s Medical Research Institute, University of Edinburgh, Edinburgh, United Kingdom; ^2^ School of Medicine, Dentistry and Biomedical Sciences, Centre for Experimental Medicine, Queen’s University of Belfast, Belfast, United Kingdom; ^3^ Laboratory of Tumour Inflammation and Angiogenesis, Department of Oncology, Leuven, Belgium; and; ^4^ Laboratory of Angiogenesis and Vascular Metabolism, Vesalius Research Centre, Leuven, Belgium

**Keywords:** IL-4, neutrophil, acute respiratory distress syndrome, hypoxia, hypoxia-inducible factor-1α

## Abstract

**Rationale:** Acute respiratory distress syndrome is defined by the presence of systemic hypoxia and consequent on disordered neutrophilic inflammation. Local mechanisms limiting the duration and magnitude of this neutrophilic response remain poorly understood.

**Objectives:** To test the hypothesis that during acute lung inflammation tissue production of proresolution type 2 cytokines (IL-4 and IL-13) dampens the proinflammatory effects of hypoxia through suppression of HIF-1α (hypoxia-inducible factor-1α)-mediated neutrophil adaptation, resulting in resolution of lung injury.

**Methods:** Neutrophil activation of IL4Ra (IL-4 receptor α) signaling pathways was explored *ex vivo* in human acute respiratory distress syndrome patient samples, *in vitro* after the culture of human peripheral blood neutrophils with recombinant IL-4 under conditions of hypoxia, and *in vivo* through the study of IL4Ra-deficient neutrophils in competitive chimera models and wild-type mice treated with IL-4.

**Measurements and Main Results:** IL-4 was elevated in human BAL from patients with acute respiratory distress syndrome, and its receptor was identified on patient blood neutrophils. Treatment of human neutrophils with IL-4 suppressed HIF-1α–dependent hypoxic survival and limited proinflammatory transcriptional responses. Increased neutrophil apoptosis in hypoxia, also observed with IL-13, required active STAT signaling, and was dependent on expression of the oxygen-sensing prolyl hydroxylase PHD2. *In vivo*, IL-4Ra–deficient neutrophils had a survival advantage within a hypoxic inflamed niche; in contrast, inflamed lung treatment with IL-4 accelerated resolution through increased neutrophil apoptosis.

**Conclusions:** We describe an important interaction whereby IL4Rα-dependent type 2 cytokine signaling can directly inhibit hypoxic neutrophil survival in tissues and promote resolution of neutrophil-mediated acute lung injury.

**At a Glance Commentary**

**Scientific Knowledge on the Subject**

The role for type 2 cytokines, such as IL-4 and IL-13, in inflammation resolution has been extensively studied, but their precise effect on neutrophils in this context remains largely unknown. Neutrophils are key innate cells adapted to working under severe hypoxic conditions, an environment that drives their survival. Such conditions are found in the lungs of patients with acute respiratory distress syndrome.

**What This Study Adds to the Field**

In this article we identify a significant role for IL-4 and IL-13 as regulators of neutrophil hypoxic survival in both human and mouse cells using a combination of *in vitro* and *in vivo* studies. We demonstrate that IL-4 is present in the lungs of acute respiratory distress syndrome sufferers and in mouse models of lung injury. Furthermore, IL-4 abrogates neutrophil hypoxic survival *in vitro* in human and mouse neutrophils in a prolyl hydroxylase 2–dependent manner. Finally, through the careful use of animal models including chimera studies we demonstrate the therapeutic potential of manipulating this pathway in the lung during hypoxic neutrophilic lung inflammation.

Acute respiratory distress syndrome (ARDS) is a clinical syndrome defined by the presence of bilateral pulmonary infiltrates and arterial hypoxemia. Although several causes are recognized including sepsis, trauma, and aspiration, ARDS is the consequence of a disordered inflammatory response in which accumulation of activated neutrophils within the lung interstitium and distal airspaces is associated with lung endothelial and epithelial injury. A fine balance therefore exists between an effective innate response that enables host pathogen control and a disordered one in which inappropriate neutrophil persistence and activation drives tissue injury. Production of local factors during the course of an inflammatory response together with changes in the physiologic environment, are crucial in regulating this balance.

Hypoxia frequently characterizes acute inflammatory sites (1) and is an important mediator of neutrophil survival and inflammatory function (2). In human disease states, hypoxia has been associated with elevated neutrophil numbers, reduced neutrophil apoptosis, and increased disease severity (3–6). Thus, hypoxia can be regarded as a proinflammatory stimulus that facilitates the persistence of neutrophils at inflamed sites.

*In vivo* models have shown that enhanced neutrophil survival in hypoxia is dominantly mediated by stabilization of the transcription factor HIF-1α (hypoxia-inducible factor-1α) (7–9). HIF-1α itself is regulated by a family of dioxygenases including the prolyl hydroxylase domain containing enzymes (PHD1–3) and FIH (factor inhibiting HIF) (10). Other studies have delineated oxygen-independent regulation of the HIF pathway, with bacterial activation of toll-like receptor signaling pathways leading to HIF-1α stabilization (11, 12). The interaction between hypoxia and local factors in regulating HIF-1α signaling, and the consequences for inflammatory responses, are therefore of great interest. IL-4 has long been considered an important cytokine for tissue repair, counterbalancing the effects of proinflammatory type 1 cytokines on the macrophage compartment (13), but conversely for promoting allergic airway inflammation. Interestingly, mouse neutrophils express both IL4Rα and IL13Rα (14), the components of the type 2 IL-4 receptor complex, through which both IL-4 and IL-13 signal. IL4Rα is upregulated in myeloid cells after stimulation with proinflammatory mediators, including LPS, with neutrophils displaying the highest fold increase in receptor expression (15). Moreover, IL-4 is itself released by macrophages after LPS simulation during the acute response (16). With cell-specific consequences of IL-4 signaling, we questioned whether IL-4 could regulate neutrophilic inflammation during acute lung inflammation to promote inflammation resolution and restoration of tissue homeostasis. We provide evidence of activation of IL-4 pathways in the setting of acute lung injury (ALI) and demonstrate that IL-4 prevents neutrophil hypoxic HIF-1α induction, abrogates hypoxic survival of human and mouse neutrophils, dampens proinflammatory cytokine expression, and promotes resolution of neutrophilic inflammation *in vivo*.

## Methods

### Blood Donors for Isolation of Human Peripheral Blood Neutrophils

Participants were consented in accordance with the Declaration of Helsinki principles, with Accord Medical Research Ethics Committee approval for study of healthy human volunteers through the Medical Research Council/University of Edinburgh Centre for Inflammation Research blood resource (15-HV-013).

### ARDS Patient Samples

BAL samples were studied from patients recruited to the HARP study (06/NIR02/77) and healthy control subjects undergoing research bronchoscopy (12/NI/0082) (*see* the online supplement). Blood samples were obtained from patients recruited to the METACYTE study (17/SS/0136/AM01).

### Animals

*Il4ra*^*−/−*^ mice on a C57BL/6 background were generated by Dr. Frank Brombacher following backcross of the BALB/*Il4ra*^*−/−*^ strain (minimum nine generations) (17) and a gift from Dr. Cecile Benezech. Animal experiments were conducted in accordance with the UK Home Office Animals (Scientific Procedures) Act of 1986 with local ethical approval.

### Human Neutrophil Isolation and Culture

Human blood neutrophils, isolated by dextran sedimentation and discontinuous Percoll gradients, were cultured (5 million cells/ml) in normoxia (21% O_2_, 5% CO_2_) or hypoxia (1% [3 kPa] O_2_, 5% CO_2_) ± recombinant human IL-4 (10 ng/ml), IL-13 (100 ng/ml) or IFN-γ (100 ng/ml; Peprotech), LPS (100 ng/ml; R515; Enzo), IL4Rα (polyclonal blocking antibody; R&D Systems), STAT3 (200 nM 5,15-DPP; Sigma), or STAT6 (20 nM AS 1517499; Axon Medchem).

### Murine Neutrophil Culture

Inflammatory neutrophils isolated by BAL 24 hours after LPS challenge were cultured at 2 million cells/ml as detailed previously.

### Chimera Studies

To dissect the consequence of IL4Ra loss on bone marrow and circulating neutrophil populations in the context of hypoxia, we used an organ-protected chimeric approach in the liver (regional hypoxia) and a fractionated radiation strategy in the lung (systemic hypoxia). Inflammation outcomes were defined in a model of acute liver injury (CCl_4_) and ALI (LPS) (*see* online supplement).

### IL4c Experiment

A total of 50 μl IL4c (250 μg/ml recombinant murine IL-4 [Peprotech]: 1,250 μg/ml antimouse IL-4 [clone 11b11; Bio X Cell]) or PBS vehicle control was administered intratracheally 6 hours after LPS-nebulization and mice transferred to hypoxia.

### Statistical Analysis

For nonparametric data significance was determined by Mann-Whitney *U* test. Data shown as individual points with median and interquartile range values. For multiple comparisons with one variable we used one-way ANOVA with Kruskal-Wallis *post hoc* test and for two variables, two-way ANOVA with Holm-Sidak *post hoc* test analysis, and adjustment for multiplicity of tests. Data shown as mean ± SEM. *P* less than 0.05 was considered significant. Individual data points represent individual mice or human samples.

## Results

### IL-4 Receptor α Signaling Pathways Are Present in the Airways of Patients with ARDS, Circulating Neutrophils, and in a Mouse Model of ARDS

**Figure 1.**
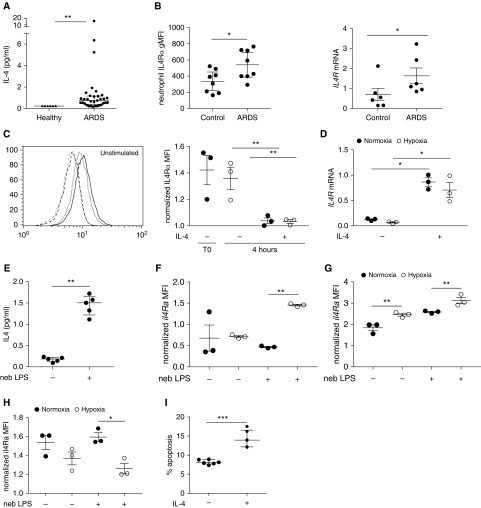
IL-4 is present in human and mouse models of acute respiratory distress syndrome (ARDS) with IL-4Ra expression found in both human and mouse neutrophils. (*A*) IL-4 levels were measured in BAL samples obtained from patients with ARDS and healthy control subjects by ultrasensitive ELISA. (*B*) Neutrophils from whole blood were identified by flow cytometry from patients with ARDS and healthy control subjects and levels of IL-4Ra surface protein and transcript measured. (*C*) Representative histogram (dashed black line = normoxia isotype, dashed gray = hypoxia isotype, black solid line = normoxia, gray solid line = hypoxia) and summary data of IL4Rα expression on human peripheral blood neutrophils after 4 hours of culture with or without IL-4. (*C*) Cell surface protein was determined by flow cytometry, with freshly isolated (T0) cells for comparison, and (*D*) mRNA was quantified relative to *ACTB* by qRT-PCR. (*E*) IL-4 levels from BAL from LPS-treated mice or naive mice (C57BL/6) obtained at 24 hours was measured by multiplex assay and (*F*) levels of IL-4Ra expression on lung neutrophils, (*G*) alveolar macrophages, and (*H*) T cells were measured by flow cytometry. (*I*) Apoptosis rates of inflammatory BAL neutrophils harvested from normoxic C57BL/6 mice 24 hours after LPS nebulization (neb) and then cultured *ex vivo* for 6 hours in hypoxia with or without 100 ng/ml IL-4 as determined by morphology. Data are expressed as individual data points with median and interquartile range (*A*, *B*, *E*, and *I*) or mean ± SEM (*C*, *D*, and *F*–*H*). Statistical significance was determined by Mann-Whitney *U* test (*A*, *B* [*IL4R* mRNA], *E*, and *I*), unpaired Student’s *t* test (*B* [IL4Rα gMFI]), or two-way ANOVA with Holm-Sidak *post hoc* tests comparing each condition with unstimulated control within normoxic and hypoxic groups, respectively (*C*, *D*, *F*, and *H*). *P < 0.05, **P < 0.01, and ***P < 0.001. gMFi = geometric mean fluorescence intensity.

To determine whether patients with ARDS have evidence of local production of IL-4, we measured IL-4 levels in BAL samples of patients with ARDS compared with healthy control subjects. ARDS BAL fluid contained significantly more IL-4 than lavage samples obtained from healthy airspaces (Figure 1A). Interestingly, preliminary data suggest that neutrophils from patients with ARDS may have higher levels of IL4Ra expression than healthy control subjects (Figure 1B) supporting the relevance of this pathway to ARDS.

To confirm the ability of circulating neutrophils to respond to IL-4 in the context of physiologic hypoxia, we examined neutrophil expression of IL4Rα. Healthy human blood neutrophils demonstrated IL4Rα surface protein expression and transcript when cultured in normoxia (21% O_2_) and hypoxia (1% O_2_), with loss of surface protein and induction of mRNA after stimulation with IL-4 (Figures 1C and 1D).

To determine if the IL-4 pathway is activated in an ALI mouse model we examined IL-4 BAL levels from mice exposed to nebulized LPS. In keeping with the data from human ARDS BAL, IL-4 is produced in the airspace of mice exposed to LPS (Figure 1E). We then determined if systemic hypoxia (10% Fi_O_2__) altered the neutrophil inflammatory responses *in vivo* and found that *in vivo* lung neutrophils express the IL4Ra, more so in hypoxia than in normoxia, after LPS (Figure 1F). In this setting alveolar macrophages also upregulate their IL4Ra expression in hypoxia after LPS stimulation (Figure 1G) but T cells do not (Figure 1H). Importantly, BAL inflammatory neutrophils cultured *ex vivo* in hypoxia with IL-4 had reduced hypoxic survival thus suggesting IL-4 can overcome the enhanced neutrophil survival observed in physiologic and pathophysiologic hypoxia (Figure 1I).

### IL-4 Abrogates Neutrophil Hypoxic Survival and Suppresses Proinflammatory Responses

**Figure 2.**
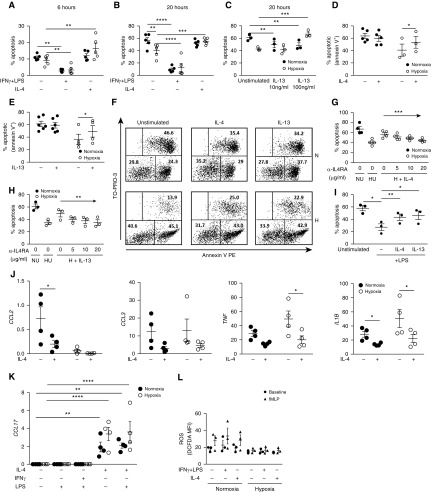
Modulation of human neutrophil hypoxic survival and inflammatory responses by IL-4. (*A–F*) Apoptosis of cytokine-treated neutrophils was assessed by morphology at 6 (*A*) and 20 hours (*B* and *C*) or by flow cytometry with annexin V at 20 hours (*n* = 3–6) (*D*–*F*). (*G* and *H*) Neutrophil apoptosis after preincubation with IL4Rα-neutralizing antibody (α-IL4RA) before culture with IL-4 or IL-13 for 20 hours in hypoxia (*n* = 3–4). (*I*) Effect of IL-4 and IL-13 on LPS-induced survival in normoxia at 20 hours (*n* = 3) as determined by morphology. (*J*) Effects of IL-4 on LPS-mediated proinflammatory gene induction in human peripheral blood neutrophils cultured for 4 hours in normoxia or hypoxia, determined by qRT-PCR (relative to *ACTB*) (*n* = 4). (*K*) *CCL17* expression relative to *ACTB* was determined by qRT-PCR, after 4 hours of culture in normoxia or hypoxia in the presence of IFN-γ, IL-4, and/or LPS (*n* = 4). (*L*) Reactive oxygen species (ROS) production was determined by flow cytometry using the fluorescent oxygen sensor DCF-DA. Neutrophils were incubated with IL-4 or IFN-γ + LPS for 4 hours with or without 100 nM *N*-formyl-met-leu-phe (fMLP) for a further 45 minutes (*n* = 4). Data are expressed as mean ± SEM. Significance was determined by repeated measures two-way ANOVA with Holm-Sidak *post hoc* test comparing cytokine treatments with unstimulated control within the normoxic and hypoxic groups, respectively (*A*–*E*, and *K*), or LPS control (*J*), repeated measures one-way ANOVA with *post hoc* test for linear trend (*G* and *H*) or Holm-Sidak *post hoc* test (*I*), or separate two-way ANOVAs for baseline and fMLP-stimulated ROS production, with Holm-Sidak *post hoc* tests comparing IFN-γ + LPS and IL-4 with unstimulated control within normoxic and hypoxic groups, respectively (*L*). *P < 0.05, **P < 0.01, ***P < 0.001, and ****P < 0.0001. HU = hypoxia unstimulated; NU = normoxia unstimulated; PE = phycoerythrin.

We next sought to determine whether IL-4 modified human neutrophil apoptosis *in vitro*. In marked contrast to the prosurvival effects of IFN-γ/LPS, IL-4 had no effect on constitutive apoptosis in normoxia, but inhibited hypoxic survival at both early (6 h) (Figure 2A) and late (20 h) (Figure 2B) time points, as determined by morphologic appearance (chromatin condensation and loss of characteristic lobes and bridges). Culture with IL-13, which also signals through IL4Rα, similarly resulted in loss of hypoxic neutrophil survival (Figure 2C). Abrogation of hypoxic survival by IL-4 (Figures 2D and 2F) and IL-13 (Figures 2E and 2F) was further validated by flow cytometry. We confirmed that these effects were IL4Rα-dependent using an IL4Rα blocking antibody, which restored hypoxic survival in the presence of IL-4 and IL-13 (Figures 2G and 2H). Abrogation of enhanced neutrophil survival was not confined to hypoxic responses, with IL-4 and IL-13 also partially reversing increased survival after LPS stimulation (Figure 2I). In addition to its effects on neutrophil hypoxic survival, IL-4 suppressed LPS-induced expression of the proinflammatory genes *CCL2*, *CCL3*, *TNF*, and *IL1B*, previously linked to HIF-1α activity (Figure 2J) (18). Conversely, IL-4 induced *CCL17* (Figure 2K), a chemokine associated with Treg recruitment, M2-polarized macrophages, and tumor-associated neutrophils (19–22). IL-4 did not influence baseline reactive oxidative species production or neutrophil respiratory burst after stimulation with *N*-formyl-met-leu-phe under normoxic or hypoxic conditions (Figure 2L).

### IL-4 Suppresses Hypoxic Induction of Nuclear Factor-κB Protein Despite Preserved Metabolic Capacity

**Figure 3.**
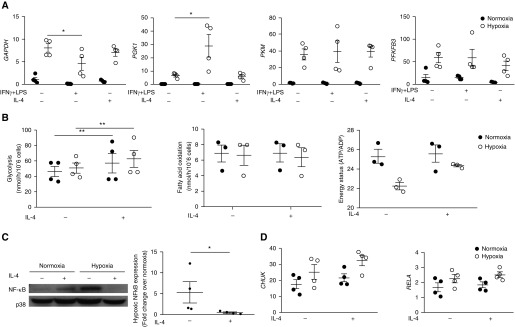
IL-4 regulates NF-κB (nuclear factor-κB) at a post-transcriptional level without altering metabolic flux. (*A*) Expression of glycolytic genes in human neutrophils after 8 hours of culture in normoxia or hypoxia with IL-4 or IFN-γ + LPS, determined by qRT-PCR relative to *ACTB* (*n* = 4). (*B*) Flux through glycolysis and fatty acid oxidation were quantified by ^3^H_2_O release after uptake of [5-^3^H]-glucose and [9,10-^3^H]-palmitic acid, respectively, in normoxia or hypoxia with or without IL-4. Energy status (ATP/ADP ratio) after 4 hours was determined by reverse phase HPLC (*n* = 3). (*C*) NF-κB p65 (RELA) protein levels after 6 hours of culture in normoxia or hypoxia with or without IL-4 were assessed by Western blot, levels quantified relative to p38 by densitometry, and fold change calculated relative to normoxia (*n* = 4). (*D*) Gene expression of *RELA* (NF-κB) and *CHUK* (IKKα) relative to *ACTB* was determined by qRT-PCR after 4 hours of culture in normoxia or hypoxia with or without IL-4 (10 ng/ml) (*n* = 4). Data are expressed as mean ± SEM. Statistical significance was determined by repeated measures two-way ANOVA with Holm-Sidak *post hoc* test comparisons with unstimulated control subjects within the normoxic and hypoxic groups, respectively (*A*, *B*, and *D*) or Mann-Whitney *U* test (*C*). *P < 0.05 and **P < 0.01.

Given that hypoxia upregulates glycolysis (2) and IL-4 has been shown to affect macrophage metabolism (23) we sought to determine whether the effects of IL-4 on neutrophil survival were through alteration of metabolic pathways. *GAPDH*, *PKM*, *PGK1*, and *PFKFB3* levels were, however, unaltered after IL-4 treatment *in vitro* (Figure 3A). Although a modest decrease in glucose uptake was observed with IL-4 (*see* Figure E1A in the online supplement), overall neutrophil glycolytic capacity was preserved (Figure 3B), in contrast to the described effects in macrophages (23).

In macrophages, polarization of phenotypic states has been linked to metabolic rewiring, with IL-4 favoring fatty acid oxidation (23). IL-4 did not affect neutrophil expression of *PPARGC1B*, a key regulator of β-oxidation (*see* Figure E1B) or flux through fatty acid oxidation (Figure 3B). Overall energy status (ATP/ADP ratio) was also not modified by IL-4 (Figure 3B), suggesting that altered metabolism and/or a lack of ATP are not responsible for the reduction in *in vitro* neutrophil survival.

We have previously shown NF-κB (nuclear factor-κB) upregulation to be critical for enhanced neutrophil survival under hypoxic conditions (8). *Hif1a*^−/−^ neutrophils have reduced expression of the NF-κB subunit *Rela* and of *Ikka*, a kinase that targets the NF-κB inhibitor IκB for degradation. We found that IL-4 diminished NF-κB RelA protein expression under hypoxic conditions (Figure 3C). However, this regulation occurred at a post-transcriptional level, with the relative expression of *RELA* and *CHUK* (IKKα) mRNA unchanged by stimulation with IL-4 in the setting of hypoxia (Figure 3D).

### IL-4–induced STAT Signaling Leads to Loss of Hypoxic Survival by Induction of PHD2 and Degradation of HIF1α Protein

**Figure 4.**
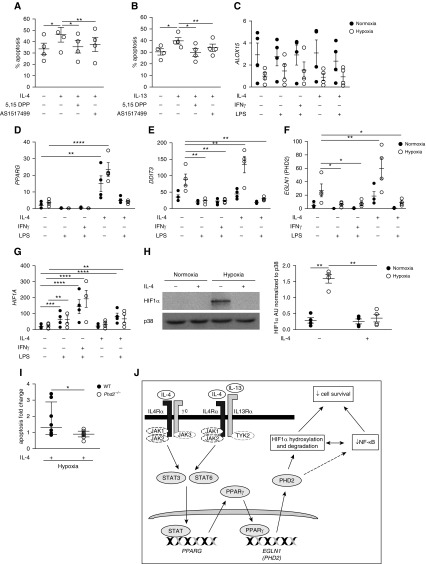
IL-4–mediated STAT/PPARγ signaling upregulates PHD2 transcript and reduces HIF-1α (hypoxia-inducible factor-1α) protein levels, resulting in loss of hypoxic neutrophil survival. (*A* and *B*) Effect of STAT3 and STAT6 inhibitors (5,15-DPP and AS 1517499, respectively) on cytokine-induced human neutrophil apoptosis in hypoxia at 20 hours (determined by morphology) (*n* = 3–4). (*C*–*E*) Neutrophil expression of genes involved in PPARγ signaling and (*F*) of *EGLN1*/*PHD2*, after 4 hours of cytokine/LPS treatment in normoxia or hypoxia, determined by qRT-PCR (relative to *ACTB*) (*n* = 4). (*G*) *HIF1A* gene expression, after 4 hours of cytokine/LPS treatment in normoxia or hypoxia, determined by qRT-PCR (relative to *ACTB*) (*n* = 4). (*H*) HIF-1α protein levels after 6 hours of culture with IL-4 in normoxia or hypoxia were assessed by Western blot and levels relative to p38 quantified by densitometry (*n* = 4). (*I*) *Phd2*^*−/−*^ mice or their littermate control subjects were treated with nebulized LPS and BAL neutrophils treated with IL-4 (100 ng/ml) in hypoxia *ex vivo* for 6 hours. Data normalized to apoptosis rates in unstimulated hypoxia control. (*J*) Illustration of IL-4 signaling pathway leading to loss of hypoxic survival in neutrophils. Data are expressed as mean ± SEM (*A*–*H*) or median and interquartile range (*I*). Statistical significance was determined by one-way ANOVA with Holm-Sidak *post hoc* test comparing cytokine-only group with every other group (*A* and *B*), repeated measures two-way ANOVA with Holm-Sidak *post hoc* test comparisons with unstimulated control subjects within normoxic and hypoxic groups, respectively (*C*–*H*), or Mann-Whitney *U* test of log-transformed data (*I*). *P < 0.05, **P < 0.01, ***P < 0.001, and ****P < 0.0001. NF-κB = nuclear factor-κB; WT = wild-type.

Previous studies have linked IL-4 signaling through IL4Rα to activation of STAT6 pathways and protection in inflammatory models of arthritis (24, 25). In macrophages, STAT3 and STAT6 are important mediators of IL-4 and IL-13 signaling through IL4Rα/γc and IL4Rα/IL13Rα (26). *In vitro* preincubation with either the STAT3 inhibitor 5,15-DPP or the STAT6 inhibitor AS1517499 was sufficient to rescue hypoxia-induced survival in the presence of IL-4 or IL-13 (Figures 4A and 4B), suggesting both STAT3 and STAT6 are required for IL-4 to modulate neutrophil survival. Neither 5,15-DPP nor AS1517499 affected hypoxic neutrophil survival in the absence of exogenous cytokine stimulation (*see* Figure E2A).

STAT6-mediated enhancement of PPARγ activity (27) and STAT3/6-mediated induction of transcripts for both *PPARG* and *ALOX15* (encoding an enzyme that catalyses production of endogenous PPARγ agonist) has previously been observed with IL-4 stimulation in macrophages (26). In contrast to macrophages, neutrophil *ALOX15* transcript was downregulated by hypoxia and unaffected by IL-4 (Figure 4C), but *PPARG* transcript was strongly upregulated by IL-4 in both normoxia and hypoxia (Figure 4D). PPARγ has been shown to induce gene expression of a “master regulator” of apoptosis, DNA damage inducible transcript 3 (DDIT3) (28, 29). Although IL-4 had an effect on DDIT3 expression, this was only in addition to the effect seen in hypoxia (Figure 4E). PPARγ has also been directly linked to increases in PHD transcript and protein abundance during adipocyte differentiation (30). We questioned whether IL-4 stimulation *in vitro* could lead to a paradoxical increase in PHD activity and thereby suppress HIF. We observed that IL-4 selectively induced *PHD2* (Figure 4F), but not *PHD1* or *PHD3* transcript (*see* Figure E2B). Neither hypoxic conditions nor IL-4 affected neutrophil *HIF1A* transcript (Figure 4G). However, in keeping with PHD-mediated degradation of HIF-1α, IL-4 dramatically reduced hypoxic stabilization of HIF1α protein (Figure 4H). To directly address the role of PHD2 in the IL-4 response, airspace neutrophils from mice deficient in myeloid PHD2 were isolated. PHD2-deficient neutrophils treated with IL-4 displayed preserved hypoxic survival confirming that PHD2 expression is essential for the IL-4–induced effects on neutrophil hypoxic survival (Figure 4I).

An overview of our proposed mechanism for IL-4/IL-13–mediated reduction in hypoxic survival is shown in Figure 4J.

### Direct IL-4 Signaling Reduces Neutrophil Survival in Damaged Liver

**Figure 5.**
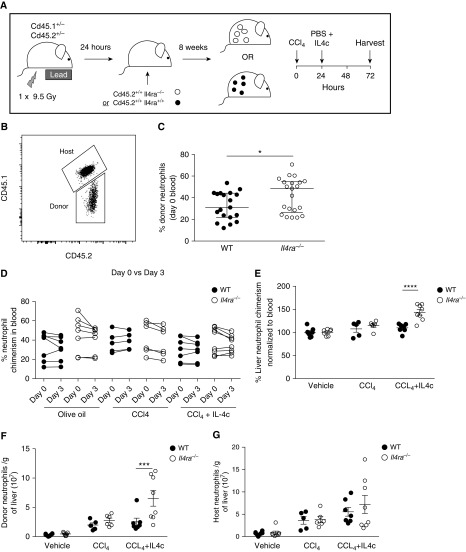
*Il4ra*^*−/−*^ neutrophils have a competitive advantage in bone marrow chimeras in a liver injury model. (*A*) *Cd45.2*^*+/−*^ *Cd45.1*^*+/−*^ mice were irradiated to deplete host bone marrow, then reconstituted with *Cd45.2*^*+/+*^ *Cd45.1*^*−/−*^ donor marrow, either wild-type (WT) or *Il4ra*^*−/−*^, as depicted. Mice were exposed to a single dose of CCl_4_ to induce liver damage, or olive oil vehicle control. (*B*) Chimerism (% CD45.1^−^ donor neutrophils) was determined by flow cytometry. (*C*) Blood chimerism at Day 0 (pre-CCl_4_) in mice receiving WT or *Il4ra*^*−/−*^ marrow. (*D*) Blood chimerism at Day 0 (pre-CCl_4_) and Day 3 in mice treated with CCl_4_ with or without IL4c and in olive oil vehicle control subjects. (*E*) Liver chimerism normalized to blood at Day 3 after CCl_4_. Total numbers of donor (*F*) and host (*G*) neutrophils per gram of liver at Day 3 after CCl_4_. Statistical significance was determined by unpaired Student’s *t* test (*C*), multiple *t* tests with Holm-Sidak correction (Day 0 vs. Day 3 for each treatment/genotype combination) (*D*), or two-way ANOVA with Holm-Sidak *post hoc* tests (WT vs. *Il4rα*^*−/−*^) (*E*–*G*). Data shown as individual mice with mean ± SEM. *P < 0.05, ***P < 0.001, and ****P < 0.0001.

To address whether IL4Rα-deficient neutrophils have an intrinsic survival advantage *in vivo*, we created liver-protected competitive bone marrow-chimeras. Recipient *Cd45.2*^+/−^ *Cd45.1*^+/−^ wild type (WT) mice were partially depleted of host bone marrow by hind-leg irradiation to retain resident liver immune cell populations, before receiving *Cd45.2*^*+/+*^ *Cd45.1*^*−/−*^ *Il4rα*^*−/−*^ (*Il4ra*^−/−^) or *Cd45.2*^*+/+*^ *Cd45.1*^*−/−*^ *Il4rα*^+/+^ (WT) bone marrow (Figure 5A). We used a CCl_4_-mediated acute liver injury model that causes hypoxic zone 3 hepatocytes to preferentially undergo necrosis (31) leading to neutrophil recruitment (32). We also investigated the role of exogenous IL-4 in neutrophil survival in this system. Although the percentage of blood neutrophils derived from donor CD45.1^−^ bone marrow before injury (Figure 5B) was marginally greater in mice that received *Il4ra*^*−/−*^ cells compared with those that received WT (Figure 5C), chimerism in blood neutrophils at Day 3 remained unchanged irrespective of treatment, demonstrating no additional competitive neutrophil survival advantage to IL4Rα expression in blood neutrophils after treatment (Figure 5D). Liver neutrophil chimerism was equivalent to blood chimerism in all groups except in CCl_4_-injured mice treated with IL4c, in which *Il4ra*^*−/−*^ donor neutrophils displayed a significant advantage compared with their WT counterparts (Figure 5E). This change in chimerism was caused by an increased number of donor *Il4rα*^−/−^ neutrophils in the liver of CCl_4_ + IL4c–treated mice (Figure 5F), rather than a decrease in host WT cells (Figure 5G), thereby confirming the competitive advantage conferred on *Il4rα*^−/−^ tissue neutrophils when present in a necrotic environment permeated by IL-4.

### IL4Rα-Deficient Neutrophils Demonstrate a Selective Advantage in an LPS-induced Lung Inflammation Model with Systemic Hypoxia

**Figure 6.**
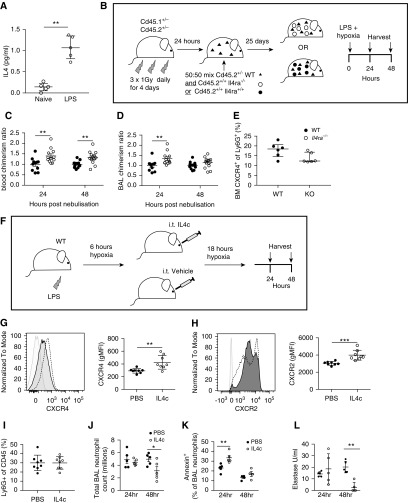
Exogenous IL-4c treatment accelerates the resolution of neutrophilic lung inflammation in hypoxia. (*A*) IL-4 levels from BAL from naive mice or mice treated with nebulized LPS and placed in hypoxia (10%) for 24 hours was measured by multiplex assay. (*B*) *Cd45.2*^*+/−*^ *Cd45.1*^*+/−*^ mice were irradiated to deplete host bone marrow (BM), then injected with donor marrow comprising 50% wild-type (WT) *Cd45.2*^*+/−*^ *Cd45.1*^*+/−*^ mixed with either 50% *Il4ra*^*+/+*^ *Cd45.2*^*+/+*^ Cd45.1^−/−^ (WT) or 50% *Il4ra*^*−/−*^ *Cd45.2*^*+/+*^ *CD45.1*^*−/−*^ (*Il4ra*^*−/−*^), as depicted. Mice were nebulized with LPS and placed in hypoxia 25 days after transplant. Neutrophil chimerism (% of Ly6G^+^ cells lacking CD45.1) was determined by flow cytometry. Chimerism was normalized to WT control chimerism at each time point. Blood (*C*) and BAL neutrophil chimerism (*D*) after LPS and hypoxia. (*E*) Proportion of donor (*Cd45.2*^*+/+*^ Cd45.1^−/−^) BM CXCR4^+^ neutrophils. (*F*) C57BL/6 mice were treated with intratracheal IL-4c or PBS 6 hours after LPS nebulization and returned to hypoxia for a further 18 hours. BM neutrophil expression of CXCR4 (*G*) and CXCR2 (*H*) was determined by flow cytometry (representative histograms shown: light gray = FMO control, dark gray = PBS, and dotted line = IL-4c). (*I*) Proportion of circulating neutrophils in whole-blood leukocytes. (*J*) Total BAL neutrophils were determined by flow cytometry. (*K*) Proportion of BAL apoptotic (Annexin V+) neutrophils was measured by flow cytometry. (*L*) Total BAL IgM release was measured at 24 hours. Data shown as individual points with median and interquartile range (*A*, *E*, *G*, *I*, and *L*) or mean ± SD (*C*, *D*, *J*, and *K*). Statistical significance was determined by two-way ANOVA with Holm-Sidak *post hoc* test (WT vs. *Il4ra*^*−/−*^ for each time point) (*C*, *D*, *J*, and *K*), Mann-Whitney *U* test (*A*, *E*, and *L*), or unpaired Student’s *t* test (*G*–*I*). *P < 0.05, **P < 0.01, and ***P < 0.001. gMFi = geometric mean fluorescence intensity; KO = knockout; WT = wild-type.

Having observed a competitive advantage for *Il4ra*^−/−^ neutrophils in a liver injury model in the presence of exogenous IL-4, we questioned whether endogenous cytokine could be sufficient to drive a selective advantage for IL4Rα-deficient neutrophils *in vivo* in the setting of systemic hypoxia combined with an LPS challenge. In keeping with previous reports of IL-4 release after LPS (Figure 1F) (33, 34), we also observe IL-4 release in the setting of hypoxia in our ALI model (Figure 6A). Competitive chimeras were generated to determine if a survival advantage was conferred by neutrophil IL4Rα deficiency in this model of ALI. *Cd45.2*^+/−^ *Cd45.1*^+/−^ WT recipient mice were fractionally irradiated to deplete endogenous bone marrow cells (35) but protect lung alveolar macrophages (*see* Figure E3A). The mice were subsequently reconstituted with a 50:50 mix of *Cd45.2*^+/−^ *Cd45.1*^+/−^ *Il4rα*^*+/+*^ WT and either *Cd45.2*^*+/+*^ *Cd45.1*^−/−^ *Il4rα*^*−/−*^ (*Il4ra*^*−/−*^) or *Cd45.2*^*+/+*^ *Cd45.1*^−/−^ *Il4rα*^*+/+*^ (WT) marrow (Figure 6B) to offer a direct comparison between WT and knockout donor cells. After a recovery period, mice were treated with nebulized LPS, placed in hypoxia (10% O_2_), and chimerism within the Ly6G^+^ neutrophil population determined by flow cytometry. Blood neutrophils at both time-points exhibited higher chimerism in *il4ra*^−/−^-recipient mice (Figure 6C), an effect that was also seen in the recruited BAL neutrophils (Figure 6D). In our model, CXCR4 expression in bone marrow neutrophils was not significantly different between donor groups, suggesting that CXCR4 was not responsible for the increased chimerism of *il4ra*^*−/−*^ blood and BAL neutrophils in this setting (Figure 6E).

### IL-4 Promotes Inflammation Resolution in Hypoxia through Increased Neutrophil Apoptosis

To determine whether IL-4 could directly promote resolution of neutrophilic inflammation in the context of systemic hypoxia, we used the same model of hypoxic airway inflammation with addition of sustained local release of IL-4 using IL-4 complex (IL4c) at 6 hours after LPS to allow for neutrophil recruitment to occur (Figure 6F). Bone marrow neutrophil expression of chemokines involved in bone marrow neutrophil retention (CXCR4) and release (CXCR2) (Figures 6G and 6H) (14) was initially explored. Although IL-4c did seem to increase the expression of bone marrow neutrophils expressing CXCR4 (Figure 6G), it also significantly increased the expression of CXCR2-expressing neutrophils *in vivo* (Figure 6H), with an equivalent proportion of circulating neutrophils observed between treatment groups (Figure 6I). Furthermore, peak recruitment to the alveolar space was also equal between groups at 24 hours (Figure 6J). During the resolution phase IL-4c treatment increased 24-hour BAL neutrophil apoptosis levels, with a subsequent reduction in BAL neutrophil counts at 48 hours (Figures 6J and 6K), and a reduction in extracellular elastase release measured in BAL fluid samples (Figure 6L).

## Discussion

We show that IL-4 signaling in neutrophils can regulate hypoxia-induced proinflammatory survival programs and can be further manipulated *in vivo* to promote restoration of constitutive rates of neutrophil apoptosis and inflammation resolution. This work extends the previously defined roles of IL-4 in type 2 mediated alternative reprogramming of macrophages during allergic, parasitic, wound healing, and acute inflammatory responses to now include direct regulation of hypoxic neutrophilic inflammation.

We show that in ARDS, a condition characterized by systemic hypoxia and uncontrolled neutrophilic inflammation, the IL-4 axis is activated and airspace neutrophils have the capacity to respond to this stimulus. Furthermore, we observe that IL-4, and the related cytokine IL-13, directly increase apoptosis of human neutrophils under hypoxic conditions and after LPS challenge, an effect that was replicated in murine inflammatory BAL neutrophils. Abrogation of hypoxic survival by IL-4 and IL-13 was dependent on the IL4Rα-downstream signaling mediators STAT3 and STAT6. PPARγ, a known target gene of STAT3 and STAT6, and important for macrophage polarization to IL-4 (26, 36), was also induced by IL-4 in healthy human neutrophils, both in normoxia, as described previously (37), and in hypoxia.

PPARγ upregulation has previously been linked to changes in neutrophil function in patients with glycogen storage disease type Ib (38), where circulating neutropenia and defective respiratory burst are described, alongside increased levels of HIF-1α. In these patients’ neutrophils, treatment with a PPARγ antagonist partially rescued the functional defects. In contrast, we observe that in metabolically sufficient neutrophils, IL4Rα signaling, with induction of PPARγ, results in downregulation of HIF-1α protein expression, associated with increased PHD2 expression. These findings are in line with a previous study describing effects of PPARγ on PHD transcription and protein levels in adipocytes (30). Importantly, we show that neutrophil PHD2 expression is essential to reproduce the inhibitory effects of IL-4 on hypoxic neutrophil survival, because PHD2^−/−^ neutrophils are resistant to IL-4–induced hypoxic apoptosis.

In addition, we also observed a decrease in hypoxic NF-κB RelA protein levels after IL-4 treatment. We have previously shown that NF-κB acting downstream of HIF1α is crucial for neutrophil hypoxic survival (8), whereas inhibition of RelA transcriptional activity by PPARγ has been implicated in apoptosis of monocyte-derived macrophages (39). NF-κB has been described as a critical transcriptional regulator of HIF-1α, but the changes in HIF-1α and NF-κB seen with the combination of hypoxia and IL-4 are limited to changes in protein abundance and thus post-translational in nature. This is of particular relevance given the overlap in post-transcriptional regulation of HIF-1α and NF-κB by asparaginyl and prolyl hydroxylation (40, 41).

Previous *in vivo* studies have demonstrated endogenous IL-4 release in the lung in response to LPS (33, 34) and upregulation of IL4Rα on myeloid cells in response to proinflammatory stimuli (15), raising the possibility that IL-4/IL4Rα signals are generated early in the course of inflammation to ensure timely resolution. Woytschak and colleagues (14) recently described a role for IL4c in limiting neutrophil recruitment during bacterial infections. In contrast, our work indicates an effect of IL-4 specifically on inflammation resolution, when IL4c is administered after onset of the inflammatory response. Using competitive chimeras, we show that in the inflamed liver, which is characterized by local hypoxia (31), *il4ra*^*−/−*^ neutrophils have an advantage over their WT control subjects with increased numbers after treatment with IL-4c, demonstrating a resistance to the proresolution effects of this cytokine. Furthermore, in our model of hypoxic ALI, BAL neutrophil counts after IL4c treatment were similar to control subjects during the recruitment phase, but lower after 48 hours, whereas apoptosis rates were higher. In keeping with IL-4 driving apoptosis-mediated inflammation resolution (42), we observed an increase in BAL neutrophil apoptosis levels at 24 hours with a subsequent reduction in BAL neutrophil counts at 48 hours after IL4c treatment and a reduction in BAL elastase. Blood neutrophil counts as a percentage of total leukocytes were equivalent and neutrophils from IL4c-treated animals expressed high levels of CXCR2, favoring marrow release. These data suggest that, in the context of systemic hypoxia and LPS, the major effect of locally delivered exogenous IL-4 on neutrophils was not on migration.

These data thus extend the current understanding from IL-4 influencing inflammation resolution via alternative macrophage activation to a model in which IL-4 concurrently limits the acute neutrophilic response through direct inhibition of hypoxic neutrophil survival. One key limitation of this work is the lack of evidence as to whether IL-4 supplementation, in addition to promoting accelerated inflammation resolution, can recover organ function. These are important future studies to determine the potential of therapies targeting IL-4. Such therapies would be limited to the site of tissue injury, where both IL-4 release and tissue hypoxia occur, and not compromise systemic neutrophil function or longevity. Further understanding of the broader importance of this pathway both to allergic airway inflammation and more chronic respiratory conditions typified by neutrophilic inflammation (e.g., chronic obstructive pulmonary disease) is also required particularly given the current therapeutic interest in blocking IL-4 signaling responses to limit disease progression.
